# CANGuard: An Enhanced Approach to the Detection of Anomalies in CAN-Enabled Vehicles

**DOI:** 10.3390/s25010278

**Published:** 2025-01-06

**Authors:** Damilola Oladimeji, Razaq Jinad, Amar Rasheed, Mohamed Baza

**Affiliations:** 1 Department of Computer Science, Sam Houston State University, Huntsville, TX 77340, USA; dko011@shsu.edu (D.O.); raj032@shsu.edu (R.J.); 2 Department of Computer Science, College of Charleston, Charleston, SC 29424, USA; bazam@cofc.edu

**Keywords:** Controller Area Network (CAN), CAN bus anomaly detection, CAN Intrusion Detection System (IDS), machine learning for CAN security, DoS attack detection in CAN, vehicle communication security

## Abstract

As modern vehicles continue to evolve, advanced technologies are integrated to enhance the driving experience. A key enabler of this advancement is the Controller Area Network (CAN) bus, which facilitates seamless communication between vehicle components. Despite its widespread adoption, the CAN bus was not designed with security as a priority, making it vulnerable to various attacks. In this paper, we propose CANGuard, an Intrusion Detection System (IDS) designed to detect attacks on the CAN network and identify the originating node in real time. Using a simulated CAN-enabled system with four nodes representing diverse vehicle components, we generated a dataset featuring Denial-of-Service (DoS) attacks by exploiting the arbitration feature of the CAN bus, which prioritizes high-criticality messages (e.g., engine control) over lower-criticality ones (e.g., infotainment). We trained and evaluated several machine learning models for their ability to detect attacks and pinpoint the responsible node. Results indicate that Gradient Boosting outperformed other models, achieving high accuracy in both attack detection and node identification. While the Multi-Layer Perceptron (MLP) model demonstrated strong attack detection performance, it struggled with node identification, achieving less than 50% accuracy. These findings underscore the potential of tree-based models for real-time IDS applications in CAN-enabled vehicles.

## 1. Introduction

Transportation has become an essential aspect of modern life, with more than 278 million vehicles registered in the US as of 2022, and 72% of individuals who rely on personal vehicles as their primary mode of transport [1,2]. This widespread reliance on vehicles has driven advancements in automotive technology, transforming cars from mere luxuries into indispensable tools for daily living.

A critical innovation in this domain is the development of in-vehicle communication protocols, which facilitate seamless data transfer among a vehicle’s components. Modern vehicles, equipped with 50 to 100 Electronic Control Units (ECUs), rely on these protocols to manage key systems such as the engine, brakes, and infotainment. These ECUs work together via robust communication networks to ensure efficiency and safety in vehicle operation [3].

The Controller Area Network (CAN) bus, developed by Robert Bosch in 1986 [4], is a widely adopted in-vehicle communication protocol. Its scalability, robustness, and fault detection capabilities have made it a leading serial bus system, reducing wiring complexity while ensuring efficiency [5]. Beyond vehicles, CAN bus systems are also integral to aircrafts, drones, farm machinery, and space satellites [5,6,7,8,9].

**Figure 1:**
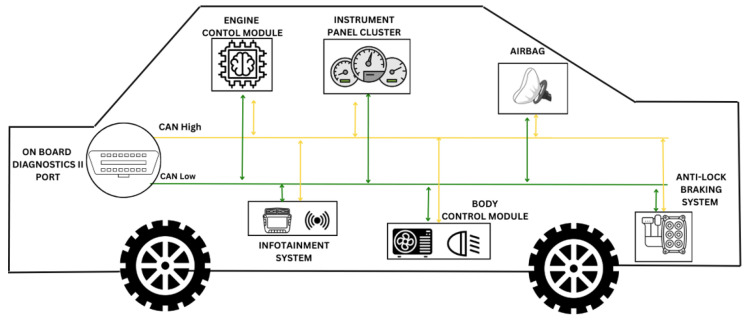
A diagram of common ECUs connected internally via CAN bus along the CAN high and low wires.

The CAN network enables communication among multiple ECUs in a vehicle through two primary wires: CAN High and CAN Low [10]. Key ECUs, such as the Engine Control Unit (ECU), Antilock Braking System (ABS), Airbag Control Module, and On-Board Diagnostic Unit (OBD), collaborate to manage and monitor critical systems, ensuring safety and performance. Figure 1 illustrates their connectivity within a CAN-enabled vehicle.

The security of modern vehicles has become a growing concern in recent years, particularly with the widespread adoption of the CAN protocol [11]. Although CAN easily allows for adding functionalities to a vehicle, increasing its complexity, this innovation also creates opportunities for attacks against these complex systems. With the advent of electronic and autonomous cars, reports of malicious attackers stealing or sabotaging vehicles have risen. For instance, in 2022, a sophisticated group of malicious actors stole Mr. Tabor’s new Toyota SUV model by targeting the CAN bus protocol [12]. They used a PIC18F chip concealed within the Bluetooth component to execute a CAN injection attack, spoofing the system by masquerading as a smart key ECU and transmitting messages to unlock the car doors and start the engine.

Aside from the Bluetooth component being used as an access point for the attacks, as in Mr. Tabor’s case, other vehicle components have also been exploited as attack entry points. Some examples of these vehicle components include the telematics control unit [13], which provides connectivity for various vehicle services and can be targeted to access the vehicle’s network; infotainment units [14], such as Bluetooth and WiFi modules; and the On-Board Diagnostics (OBD-II) port [15]. Malicious actors often target these components to attack the CAN bus, mainly due to the broadcast nature of CAN messages. Although the CAN bus offers Cyclic Redundancy Checks (CRC) to check for inconsistencies in messages sent by nodes/ECUs on the bus, there is no inherent security measure within the bus to defend against attacks. Furthermore, our previous research demonstrated that CAN-enabled systems are vulnerable to spoofing, injection attacks, and denial of service (DoS) attacks [16]. Furthermore, researchers have shown that the CAN bus is susceptible to replay attacks, impersonation attacks, fuzzy attacks [17], man-in-the-middle attacks, and bus-off attacks, among others [18]. This highlights the need for a system to detect these attacks and alert drivers or vehicle owners, ensuring immediate action is taken.

In this paper, we propose the development of a security system that can immediately detect attacks, thereby allowing the driver to take action instantly. We take it a step further by ensuring this system can also determine the part of the car that is under attack. To develop our attack detection system, we generated a dataset using a CAN-enabled testbed with four nodes, each acting as ten arbitrary nodes. In this dataset, we created scenarios that simulate injection attacks, leading to DoS attacks within the CAN network. Hence, we are proposing the development of CANGuard, a CAN Intrusion Detection System (IDS) facilitated by machine learning algorithms. An IDS monitors systems for anomalies or attacks that could lead to security breaches. We will conduct experiments to monitor the performance of different machine-learning models in detecting these anomalies or attacks. The machine learning algorithms that will be used to develop this IDS are logistic regression, random forest classifier, gradient boosting classifier, multilayer perceptron.

The structure of this paper is as follows: Section 2 provides a review of existing works on the development of IDS systems for CAN-enabled networks. Section 3 details the general architecture of the CAN network and the testbed used in this research. Section 4 describes the CAN bus threat model, common attacks the network is susceptible to, and the specific attack model adopted for this study. Section 5 outlines the methodology used to develop the proposed IDS, CANGuard. Section 6 presents the results of the evaluated models, while Section 7 discusses the findings, including the limitations of the research. Finally, Section 8 concludes the paper and suggests future directions.

## 2. Related Works

As previously stated, CAN is vulnerable to several attacks. Attacks on the CAN bus can have devastating effects when the car is in operation, including the potential for loss of human life or severe injury to drivers and passengers [19]. To address this issue, researchers have explored various approaches to anomaly detection in CAN-enabled vehicles to identify and mitigate potential attacks in real time. A promising approach is the development of IDS systems for CAN systems, particularly because this detection system does not change its operations, making it easy to adapt to the CAN network. Hence, in this section, we will discuss existing literature that focuses on developing IDS systems for CAN-enabled vehicles with varying detection modes.

When an IDS system is being developed, an important concept to be considered is the method the system adopts to detect these attacks [11,20,21]. An IDS system developed for the CAN network will monitor the system and extract the dynamic behavior of the CAN network, thereby using it as a reference to detect deviations from normal operation. Often, these detection mechanisms fall into one of these two major mechanisms: signature or anomaly-based detection.

Anomaly-based IDS systems identify intrusions by detecting deviations from normal behavior or activity patterns within the CAN network. They monitor the network for unusual patterns that deviate from established baselines, signaling potential security threats. Lampe et al. [22] developed an anomaly-based IDS system that operates through an Android application connected to the car’s Bluetooth component, which is plugged into the diagnostic port on the CAN bus. Their system evaluation was promising, showing little to no false positives when tested with real cars and publicly available attack datasets.

In contrast, signature-based IDS systems detect intrusions by matching patterns or sequences corresponding to known attack signatures stored in the IDS database. For example, Jin et al. [23] developed a promising signature-based IDS system for CAN that can applied directly to each ECU within the vehicle. They extracted signatures of various attacks that the CAN bus system is vulnerable to using real-world scenarios to detect attacks within the CAN network traffic. Similarly, Song et al. [24], and Bi et al. [25] proposed signature-based IDS for the CAN bus, relying heavily on the time interval of messages sent on the bus. In their experimentation, they recorded that when a CAN ID sends a packet, the time interval between packets should not be less than 0.2 milliseconds (ms); otherwise, the IDS records it as an attack. Likewise, Halder et al. [26] developed COIDS, a signature-based IDS system that detects intrusions in CAN-enabled systems by monitoring changes in clock offset. COIDS creates a baseline of normal clock behavior using clock offset measurements from ECUs and identifies deviations from this baseline to detect potential intrusions.

It is important to note that the implementation of IDS mechanisms in vehicles can vary. Depending on the specific application and the data type being monitored, IDS systems might be applied in various ways, such as machine-based, frequency-based, statistical-based [17], or specification-based approaches. Each method offers different advantages and focuses on different aspects of the data or behavior to detect anomalies and intrusions.

The use of machine learning models in the development of IDS systems is highly common due to their ability to detect complex patterns and anomalies in large datasets and their ability to ensure the IDS is easily adaptable to new attacks. For instance, Seo et al. [27] adopted Generative Adversarial Nets (GAN) to develop their anomaly-based IDS system, GIDS. Their system performed in the 99th percentile in detecting DoS, fuzzy, RPM, and gear attacks. While Sun et al. [28] also leveraged the machine learning model to develop their IDS, they developed an ensemble model using CNN and LSTM with attention mechanisms. Their model showed great results with an error rate of 2%. Other machine learning models that have been adopted in the development of IDS systems are CNN [29], Bayesian networks [21,30], Gradient Boosting Decision Tree (GBDT) [31], KNN, RF [32], gated recurrent unit (GRU) [33] among many others.

Conversely, other time-based IDS methods to develop either signature or anomaly-based ID systems are the CAN network IDS developed by Khan et al. [17] focused on analyzing the relationship between the attack ratio, average, and standard deviation of CAN bus data, completely dismissing the time intervals. Another by Lee et al. [34], which uses the offset ratio and time interval of ECUs to detect attacks within the bus. Additionally, More et al. [35] used a statistical approach to develop their IDS system, which relies heavily on identifying messages and standardizing message transfer times within the CAN bus. The vulnerabilities discovered during testing were reported directly to the vendor. Song et al. [24] developed an IDS system based on the time intervals of the CAN messages by capturing CAN data and simulating three types of message injection attacks. Others that also developed IDS using time interval analysis and arbitrary Identifiers assignments are [35,36].

In this paper, we propose the development of CANGuard, an IDS that will recognize anomalies within a CAN-enabled system. Our approach for developing this IDS system uses a hybrid of anomaly and signature detection mechanisms, thereby ensuring the enhanced detection of attacks. CANGuard is developed using attack datasets we generated to simulate real-life scenarios of DoS attacks, thus ensuring it performs effectively in securing the vehicle. Our IDS leverages an enhanced methodology employing various machine learning models, which we rigorously evaluate in terms of performance during training and testing on the dataset. This comparative analysis aims to identify the most effective model for anomaly detection, ultimately strengthening CAN bus security in vehicles.

## 3. System Architecture

In this section, we discuss the general architecture of the CAN network, highlighting its communication methods, physical components, and packet transfer mechanism.

The CAN network facilitates communication between various vehicle components, such as the engine control unit (ECU), transmission, airbags, and ABS, without complex wiring [10]. It operates within a robust framework of international standards, particularly ISO 11898 [4], which defines the rules for real-time and reliable communication between microcontrollers and devices, all without requiring a central host computer.

### 3.1. Physical Components of an ECU in the CAN Network

**Figure 2:**
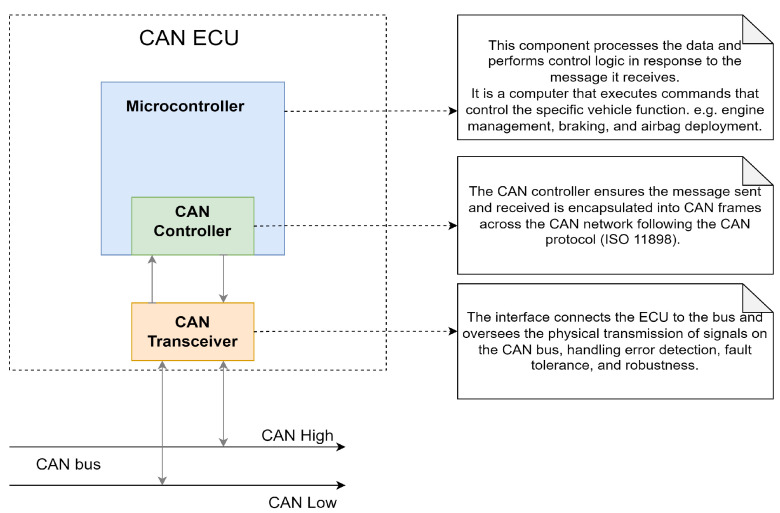
The physical components of a typical CAN ECU.

A typical ECU in a CAN-enabled vehicle consists of three key components: the microcontroller, the transceiver, and the controller. These components work together to ensure the ECU performs its tasks efficiently, enabling sensors and other parameters to function properly. This, in turn, ensures the optimal performance of the vehicle. The ISO 11898 standard underpins the communication protocols these components use, ensuring a reliable exchange of information between vehicle subsystems to maintain overall system performance. A detailed description of the functionalities of these components is highlighted and described in Figure 2.

### 3.2. CAN Bus Communication Mechanisms

One of the key advantages of the CAN bus is that it allows many of a vehicle’s components to be connected while reducing the complexity of the wiring system. Instead of using multiple wires, the CAN bus operates with just two: CAN High (CANH) and CAN Low (CANL). These two wires work together to transfer data within the bus, with each transmission representing one bit of information. CAN High (CANH) handles high-speed signals, while CAN Low (CANL) manages lower-speed signals in the network. Electromagnetic interference is minimized because the wires are twisted together and terminated with a 120-ohm resistor. All nodes or ECUs (Electronic Control Units) on the bus are connected in parallel and must be connected to CANH and CANL wires.

Modern cars often have multiple CAN networks connected via a gateway to handle the various nodes (or ECUs) within them. The high-speed CAN system (ISO 11898-2) is responsible for critical systems like ABS, airbag modules, and powertrain control units with a maximum transfer rate of signals as 1kbit - 1Mbit per second. On the other hand, the low-speed CAN bus system (ISO 11898-3) is typically used for less critical functions, such as infotainment systems (radio, Bluetooth, GPS) and door signals. Its transfer rate is 125 kbps [14]. This system works great because nodes can easily be added to the bus, and it is also easy to remove nodes from the bus.

**Figure 3:**
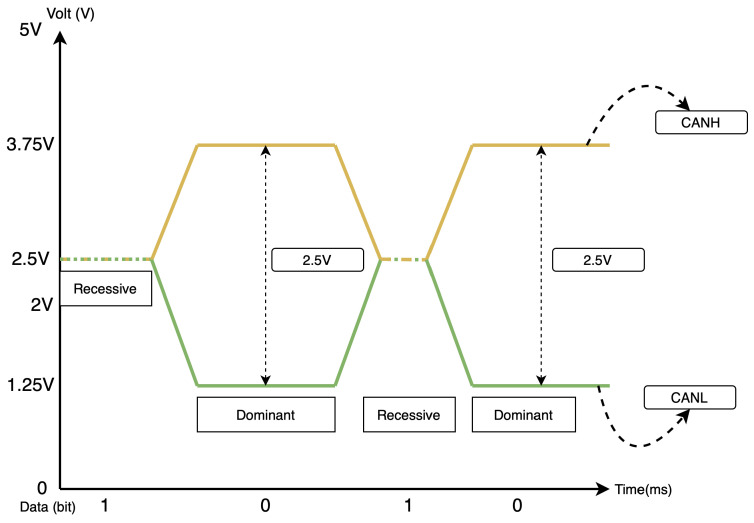
A visual illustration of the changes in the state of the bus when communication occurs.

When the CAN bus is idle (in a recessive state), the CANH and CANL lines are at 2.5 V. However, once communication or data transfer begins (in a dominant state), the voltage on these wires changes: CANH typically rises to 3.75 V, and CANL drops to 1.25 V. This creates a 2.5 V difference between the two lines, allowing data transmission to occur. Table 1 highlights the specific voltage levels of each CAN bus wire in different states. Likewise, Figure 3 gives a visual representation of the changes in the state of the bus when data is being transmitted.

### 3.3. CAN Bus Data Transfer Mechanism

When communication happens on the CAN bus, it is broadcast to all connected nodes. This means that all other nodes on the bus receive any message sent by one node. Each node then decides whether to accept or ignore the message based on whether it is relevant to its function or needs. The CAN bus ensures the integrity of messages or data sent on the bus by encapsulating them into packets called frames. Hence, we can refer to messages sent on the bus as frames. It is important to note that only one node can send a frame/packet to the bus at a time, so the CAN network is called a serial network.

Different kinds of frames/messages can be sent on the bus, and this determines the need for the node to send the packet. These various frames are:

- Data Frames: The frames contain data transfer sent by a node to be received by other nodes or ECUs on the bus.
- Remote Frames: These are initiated when a node intends to request data from other nodes.
- Error Frames: Based on the state of the bus, in the event an error occurs, these frames are used to report such errors.
- Overload frames: When the bus is overloaded, these frames report the congested state of the bus.

Irrespective of the frame sent on the bus, each frame is encapsulated with several components, each of which serves a crucial role in ensuring reliable and synchronized communication on the CAN network. These components are surveyed in Table 2.

### 3.4. CAN Bus Frame Priority (Arbitration)

The CAN bus is a serial network, meaning only one frame can be transmitted. When two or more nodes attempt to send packets simultaneously, the network uses a priority system to determine which node’s data will be transmitted. This process is known as CAN arbitration, where the message with the highest priority (lowest identifier value) is given access to the bus. At the same time, the other nodes wait until the bus is free again. This ensures that critical data is sent first. Recall the ID component encapsulated within the frame for each data transfer; this component is essential for determining the priority of the data sent on the bus. This ID helps identify how critical the data is. For example, data from the ABS will have a higher priority than data from the door signals. Since lower ID values indicate higher priority, the ABS message will have a lower numerical value than the door signal, ensuring that it is transmitted first in case both attempt to send data simultaneously.

### 3.5. The Proposed CAN Bus Testbed

To carry out this research, we developed a CAN bus testbed as the foundation for data collection, and the simulation of various attack scenarios used to develop the CAN IDS system. Our testbed consists of four physical nodes, each simulating ten ECUs. We achieved this by assigning ten random IDs unique to each node once the system was operational. As a result, the CAN network processes each message as if it came from an individual ECU, effectively simulating a system with over forty ECUs.

**Figure 4:**
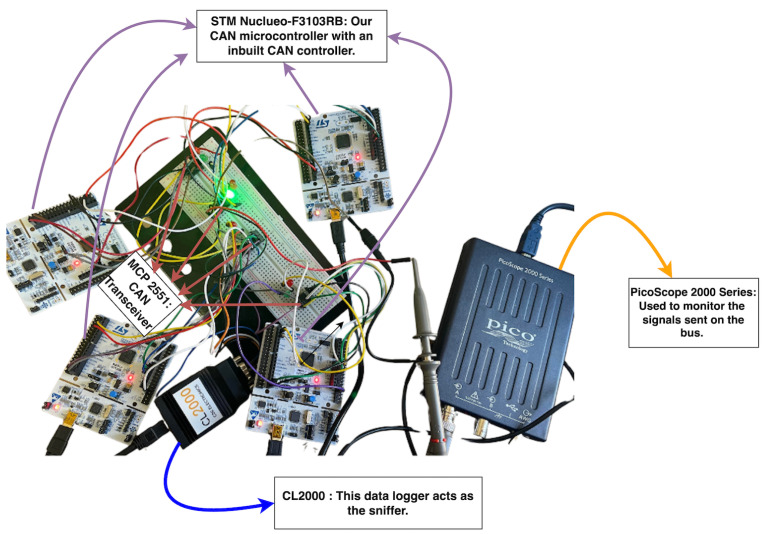
Our CAN bus enabled system.

We tested the system using a CAN data logger, which captured traffic during each run, and an oscilloscope to monitor the frames’ components transmitted within the network. Figure 4 shows the hardware components of our CAN bus testbed, and Table 3 highlights the critical components essential for its successful implementation.

#### 3.5.1. Configuration the ECU

Recall that a CAN ECU comprises of the CAN microcontroller, the CAN controller, and the CAN transceiver. Once these components are integrated into the system, configuring and testing the ECU in the network becomes straightforward.

The microcontroller was programmed with C code to program the CAN ECU using libraries compatible with the Mbed platform. Each ECU was initialized with the CAN Rx (PB_8) and CAN Tx (PB_9) pins to enable the transmission and reception of messages over the CAN bus. The message is 1 byte in size and contains an ID and a counter value. When the message is successfully transmitted, an LED lights up as a signal, and the counter is incremented for the next message. We use the CANStandard format, which specifies that the CAN frames use the standard 11-bit identifier rather than the extended 29-bit format.

Each ECU is also programmed to receive and store incoming messages. When a message is received, the ECU prints the content of the message, and another LED is turned on to indicate that a message has been successfully received. The ECU pauses for 5 milliseconds (ms) before continuing this loop, allowing for a controlled timing between operations

#### 3.5.2. System Configuration of the CAN Data Logger

**Figure 5:**
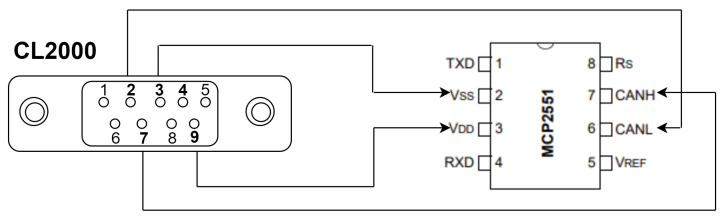
Visual illustration of the connection to the CAN network.

The CL2000 is the device we use to log data on our CAN network, functioning as a sniffer in our setup. It came preconfigured by the manufacturer, so we easily integrated it into the network by connecting pins 2, 3, 7, and 9 to our CAN transceiver on the breadboard. Table 4 highlights the connection of the CL2000 to the board. Figure 5 shows the connection of each pin to the CAN transceiver.

**Figure 6:**
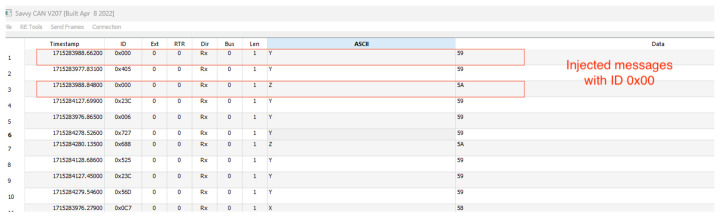
Viewing frames captured by the CANlogger using the Savvy CAN software.

To capture the traffic on the CAN bus, the connected CL2000 device records all network traffic while the CAN system is running. After each capture session, we turn off the CAN system and disconnect the CL2000 from the network. Then, we connect the CL2000 to our host computer via USB to transfer the recorded data for further analysis. After connecting the CANlogger to the host machine, we use the Savvy CAN software (version number V199) to analyze the captured traffic and export the data from the CL2000 into CSV format. An example of the frames captured by the CL2000 and viewed using the Savvy CAN tool is shown in Figure 6. Finally, we used the PicoScope 2000 series to monitor and visualize the signals transmitted on the CAN bus.

## 4. Threat Model

In this section, we will highlight some common attacks that can target the CAN network. Additionally, we will discuss the threat model used in this research and explain the attack scenarios implemented to generate the dataset used for training and developing the IDS system.

### 4.1. CAN Bus Attacks

Table 5 highlights some of the common attacks the CAN network is particularly susceptible to, further stating the effects of these attacks on the vehicle’s operations if successfully carried out.

### 4.2. CAN Bus Threat Model

After reviewing the potential attacks a vehicle with a CAN network might face, we identified the need to analyze a threat model that outlines possible approaches an attacker could exploit. We aim to simulate one of these attack paths to generate a dataset resembling real-life scenarios. This dataset will then be used to develop the IDS model implemented in this paper. The more realistic the attack, the more effectively our IDS system will perform.

We identified several key components when developing the threat model for this research. First, most attacks tend to culminate in a DoS attack, where legitimate messages from ECUs are blocked, resulting in vehicle malfunction. As a result, this research focuses on one key attack vector: injecting the CAN bus with spoofed IDs to flood the network, ultimately causing a DoS attack. By emphasizing this attack path, we aim to simulate realistic threat scenarios and develop an effective IDS system. This simulation is discussed further in Section 4.3.

**Figure 7:**
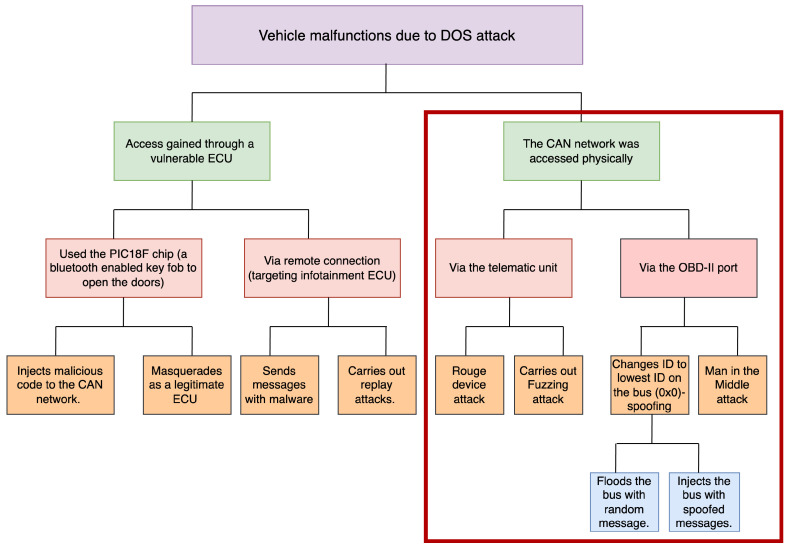
Threat model for CAN bus attacks highlighting DoS path by physical or remote access to the vehicle. The red-highlighted segment represents the attack path we simulated in this research.

In Figure 7, we illustrated the possible attack paths an attacker could use to carry out DoS attacks on a vehicle operating with the CAN network. While these attacks may not be easily executed, they can be achieved through injection, spoofing, replay, fuzzing, and MitM attacks. In the figure, we highlighted the right portion of the threat model in red to indicate that the attack simulated in this research falls within this category. Specifically, we conducted a physical attack by directly connecting to the bus. We monitored and captured messages (MitM), sent malformed messages to identify vulnerabilities (fuzzing), and then spoofed IDs to inject false messages, ultimately flooding the bus and triggering a DoS attack.

### 4.3. The Proposed CAN Bus Attack Model

To simulate our DoS attacks, we targeted a specific feature of the CAN bus: the arbitration process. Recall that the CAN network is a serial network, meaning only one message can be transmitted at a time. To prioritize messages from critical nodes, each message is assigned an ID. When two or more nodes attempt to send messages simultaneously, the message with the lowest ID takes priority and is transmitted first. Our attack strategy exploits this arbitration process by manipulating the IDs to disrupt normal communication and trigger a DoS attack. Subsequently, we drew up two scenarios for our attacks: randomizing the spoofed IDs and altering the injection timing of messages from the spoofed ID.

**Figure 8:**
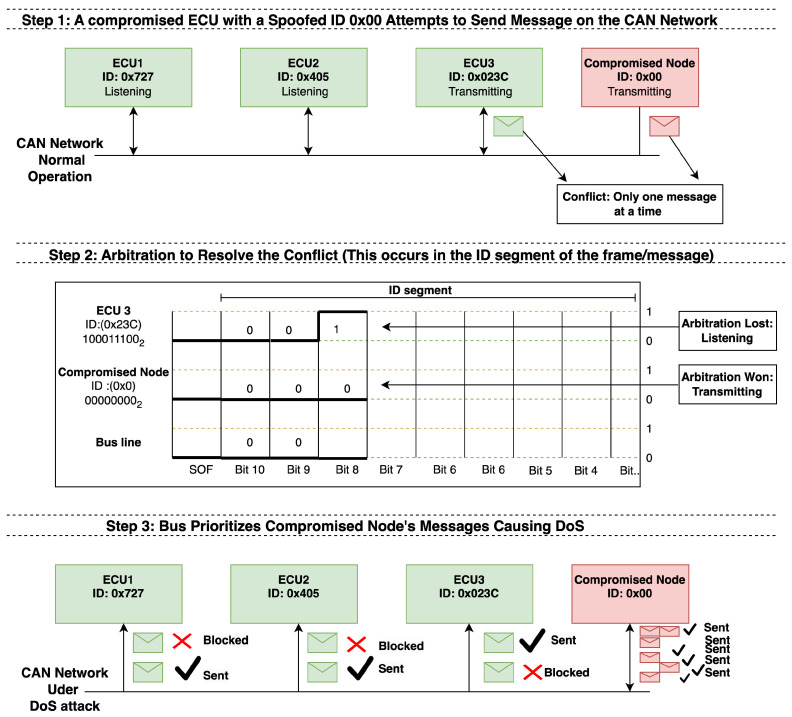
Our proposed attack model.

Our previously discussed testbed was used to carry out these attack simulations. The testbed consists of four ECUs configured to simulate the behavior of forty ECUs by encoding multiple IDs (one per ECU) into the messages during vehicle operation. To exploit the arbitration process, we spoofed the ID to 0x00, ensuring it was the lowest ID on the bus, which caused the bus to prioritize the compromised node’s messages over those from legitimate ECUs. Figure 8 provides a high-level illustration of our attack model, showing how the DoS attack was carried out.

#### 4.3.1. Attack Scenario 1: Randomizing Spoofed IDs

A crucial feature we aim for the IDS system to handle is identifying the compromised ECU’s location relative to the CAN bus. To test this capability, we developed a scenario that involves randomizing the compromised node with ID 0x00. Specifically, during the operation of the CAN-enabled system, the spoofed ID can originate from any of the four ECUs connected to the bus. By randomizing the origin of the spoofed messages, the system must not only detect that an ID has been spoofed but also identify the source ECU and its distance from the bus. The goal of this scenario is to enhance the IDS’s ability to detect and respond to dynamic, non-stationary attacks, thereby improving its overall effectiveness in securing the network.

To simulate this attack, we set up a testbed with four ECUs (ECU1, ECU2, ECU3, and ECU4). A flag triggers each compromised ECU to return to normal operation after a specific time interval of exactly 120 min. After this period, the attack shifts to the next ECU, changing its ID to 0x00, and continues for another 120 min. Once the second ECU finishes its cycle, it returns to normal operation. This process repeats sequentially across all four ECUs, ensuring that each node is affected by the attack and returns to normal operation.

After completing this eight-hour monitoring session, we collect and retrieve the log file from the CAN data logger, which is critical for gathering data. To further strengthen our testing, we randomize the data so that, within every set of 10 entries, each of the four nodes is represented as compromised. This randomized dataset is then used to train our IDS, ensuring it can effectively detect patterns across different compromised nodes. The training process is detailed in Section 5. For this scenario’s implementation, we used specific metrics, with some defined as constants and others as variables. These metrics are shown in Table 6.

#### 4.3.2. Attack Scenario 2: Randomized Injection Time

In our second scenario, we randomized the injection timing of messages sent from the compromised node. The goal of this attack is to execute a DoS attack by flooding the CAN bus with a high volume of spoofed messages. By varying the injection timing, we simulate a more sophisticated and unpredictable attack pattern, testing the IDS beyond static, predictable attacks. This variation is crucial, as real-world attackers often randomize their attack vectors to avoid detection, making time-based anomalies harder to identify.

To simulate this, we designated one ECU as the compromised node with ID 0x00. We configured the spoofed messages to be injected at irregular intervals, ranging from rapid bursts to longer delays. Specifically, the intervals were set to 1 ms, 2 ms, 3 ms, and 4 ms for this compromised node. Each time interval was monitored for two hours. After each monitoring session, the bus was powered down, and the compromised ECU was updated to the next time interval, where it was again monitored for another two hours.

Upon completing the ten-hour monitoring session, we retrieved the log file from the CAN data logger. We randomized the dataset to further enhance the robustness of the IDS system being developed. The training process for the IDS is discussed in Section 5, and the metrics for this scenario are detailed in Table 7.

## 5. Methodology of CANGuard: Our Proposed IDS

**Figure 9:**
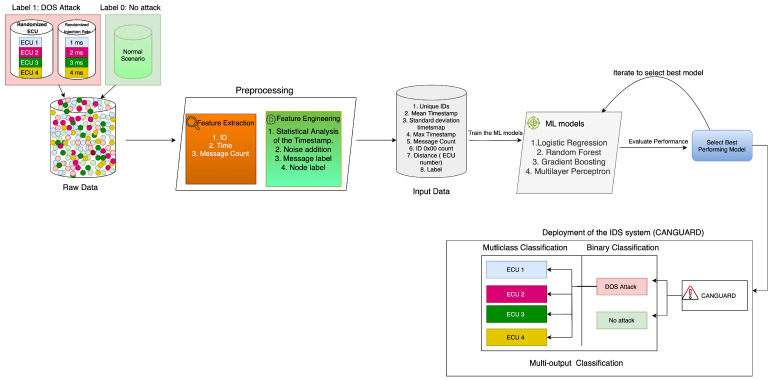
Our proposed methodology.

In this section, we outline the steps in developing CANGuard, our proposed IDS system designed to effectively and efficiently detect attacks within a CAN bus system. By leveraging advanced machine learning models, CANGuard enhances its ability to identify both known vulnerabilities and emerging threats in bus traffic, providing robust system protection. We will cover the raw dataset in Section 5.2, the data transformation steps to align with real-life scenarios, the selected machine learning models used for training and testing, and finally, the deployment of the best-performing model based on a comparison of each model’s performance in the following subsections. Figure 9 gives a high-level overview of the proposed methodology adopted in the development of the IDS system.

### 5.1. Experimental Setup

For the experimental setup to develop the IDS system, we utilized Google Colab Pro as our computational platform, leveraging its robust resources for machine learning experiments. The hardware configuration included access to a Tesla T4 GPU, which is highly effective for handling computationally intensive tasks, particularly those involving deep learning models. The system was equipped with 12.7 GB of RAM and 10 GB of GPU memory, providing sufficient resources to train and evaluate our models efficiently. Additionally, the allocated disk space of 107.7 GB allowed us to store the CAN bus dataset, feature-engineered data, and model checkpoints throughout the experimentation process. All experiments were implemented using the Python programming language, with primary reliance on the sci-kit-learn library for our machine learning models and Jupyter Notebook as the development environment.

### 5.2. Datasets

As shown in our methodology diagram, the first phase involves collecting the raw dataset. Our raw dataset was divided into two categories: normal and attack traffic. The normal dataset represents non-compromised CAN bus activity, while the attack dataset includes data from two simulated attack scenarios (Scenario 1 and Scenario 2). All datasets were collected using the CAN data logger, ensuring a consistent structure. The total size of all data collected was 7 MB. Table 8 details the dataset columns used for analysis.

The timestamp and ID columns were the key segments selected as raw data. The ID column was especially relevant in the attack scenario 1 (randomized ID) dataset, while the timestamp column was crucial in the attack scenario 2 (randomized injection rate) dataset.

### 5.3. Data Preprocessing

Data preprocessing involved feature extraction and engineering to develop a robust IDS for detecting DoS attacks. The goal was to enable the models to identify both the presence of an attack and the responsible node. Meaningful features were derived from the raw dataset to optimize model performance during training and testing.

#### 5.3.1. Feature Extraction

We used a sliding window approach to extract features from raw CAN bus data, capturing temporal patterns and distinguishing normal from attack communications. This method tracked changes in message rates, timing, and node activity within 5 ms intervals, providing essential inputs for accurate classification. Extracted features included unique ID counts, time-based metrics, message counts, error counts, and distance features.

1. **Unique IDs counts**: One of the primary features generated is the count of messages for each unique CAN ID within a 5 ms time window, labeled as ID_XX_Count, where XX represents the specific CAN ID. For each unique ID observed in the dataset, a feature tracks the frequency of its occurrences within this time frame. This feature is crucial for detecting message bursts or unusual traffic patterns, often indicators of abnormal behaviors such as those caused by a DoS attack. Monitoring message volume by specific IDs helps to identify overuse or underuse patterns, which can suggest malicious activity when they deviate from typical communication patterns.
  A particularly critical feature in detecting DoS attacks is the count of messages with ID 0x00, noted as ID_00_Count. In our DoS attack simulations, the attacking node is assigned the ID of 0x00, making this feature a direct indicator of attack activity. A significant rise in messages with this ID within the time window strongly suggests that the attacking node is attempting to flood the network, overwhelming the system and disrupting normal operations.
2. **Time-based Feature**: The timing of each message or frame is a critical factor for the IDS in identifying anomalous activity within the CAN bus network, as unusual timing patterns often signal malicious interference. To capture this, we extracted the timestamp feature, which records the precise time each message is sent. Additionally, we discuss the statistical transformations of the time-based feature in the feature engineering phase to ensure that patterns such as irregular intervals, burst frequencies, and unexpected delays can be effectively analyzed by the model.
3. **Message Count Feature**: Another important feature is the total message count within each window. This feature is particularly useful in detecting sudden surges in traffic, which are characteristic of DoS attacks. In a normal CAN bus network, the message rate remains relatively stable, whereas a DoS attack results in a sharp increase in the number of messages transmitted in a short time. Furthermore, the system also tracks the number of messages labeled as ERROR, which serves as an indicator of potential communication failures or transmission issues within the network. An increase in error messages could signify that the network is under attack, as DoS attacks often lead to message collisions, transmission errors, and other disruptions in the communication protocol.

#### 5.3.2. Feature Engineering

Feature engineering involved analyzing timestamp data and adding noise to simulate real-world network fluctuations. These engineered features enhanced the IDS’s ability to detect both subtle and overt disruptions, distinguishing normal behavior from DoS attacks while identifying their source within the CAN network. We also labeled the dataset in this phase.

1. **Statistical analysis of the Time-based feature**: We transform the raw data by carrying out statistical analysis on the time-based features extracted to capture the temporal dynamics of the CAN bus messages. The statistical analysis of the time-based feature extracted are:
  - The mean timestamp difference is calculated as the average time interval between consecutive messages within each window. This feature helps detect anomalies in the message rate, as DoS attacks tend to flood the network with a high frequency of messages, reducing the time interval between them. It is defined as
    $$\mathrm{Mean\_Timestamp\_Diff}=\frac{1}{N-1}\sum_{i=2}^{N}\left({\mathrm{timestamp}}_{i}-{\mathrm{timestamp}}_{i-1}\right)$$
  - Similarly, the standard deviation of the timestamp difference provides insights into the variability of message timing within the window. A stable network typically exhibits consistent message timing, whereas a DoS attack introduces irregularities. The standard deviation of the timestamp differences (STD) is calculated as:
    $$\mathrm{STD}=\sqrt{\frac{1}{N-2}\sum_{i=2}^{N}{\left(({\mathrm{timestamp}}_{i}-{\mathrm{timestamp}}_{i-1})-\mathrm{Mean\_Timestamp\_Diff}\right)}^{2}}$$
  - Additionally, the maximum and minimum timestamp differences were also calculated. This process further aids in capturing extreme intervals, which can also be indicative of abnormal activity. The maximum and minimum differences between consecutive timestamps are given by:
    $$\mathrm{Max\_Timestamp\_Diff}=\max_{2\leq i\leq N}\left({\mathrm{timestamp}}_{i}-{\mathrm{timestamp}}_{i-1}\right)$$
    $$\mathrm{Min\_Timestamp\_Diff}=\min_{2\leq i\leq N}\left({\mathrm{timestamp}}_{i}-{\mathrm{timestamp}}_{i-1}\right)$$
2. **Noise Addition**: Noise addition is crucial for training robust machine learning models capable of detecting DoS attacks on CAN bus networks. By simulating real-world disturbances, such as timestamp jitter, latency, packet loss, and transmission errors, we can create a more challenging and realistic training environment. Timestamp jitter introduces small, random variations in message timestamps, mimicking minor network fluctuations. Latency simulates delays in message transmission, accounting for network congestion and physical distances. Packet loss models the random loss of messages due to network errors or attacks. Transmission errors introduce corrupted messages into the dataset, mimicking the effects of hardware failures or malicious interference. By exposing the model to these diverse types of noise, we enhance its ability to distinguish between normal network behavior and malicious activity. The model learns to identify patterns that indicate a DoS attack, even in the presence of various disturbances. Ultimately, this approach leads to a more resilient and effective machine learning model for detecting and mitigating DoS attacks on CAN bus networks.
3. **Message Label**: The message label feature was created to support binary classification, which determines whether a message is normal or anomalous. Normal messages, indicating no attack, are labeled 0, while attack or anomalous messages are labeled 1.
4. **Node Label**: The node label is essential for identifying the specific node responsible for the DoS attack. During the preprocessing phase, each node in the CAN bus network is assigned a label based on its physical proximity to the central controller. This feature enables the model to determine which node is likely causing the attack. Including this feature adds a valuable layer of multi-class classification, as it not only detects the presence of an attack but also helps pinpoint its source within the network. In this case, we had four labels, where all frames from ECU1, ECU2, ECU3, and ECU4 are assigned labels 1, 2, 3, and 4 respectively.

The data preprocessing phase centers on extracting key features through a window-based approach and generating meaningful features via feature engineering techniques, resulting in a robust input dataset. For the experimental analysis, the dataset consisted of CAN bus frames, each representing a single message exchanged on the network. These frames were grouped into windows based on a sliding window approach with a size of 5000 milliseconds (5 s). Within each window, statistical features were computed, such as message counts, timestamp differences, and error counts, providing a summarized representation of the network activity during that period. The total number of windows generated depended on the number of frames in the dataset and the chosen window size. For training and testing, the windows were split into an 80-20 ratio, ensuring a balanced distribution of attack and non-attack scenarios across the datasets. By preprocessing the data in this way, the system can effectively detect anomalies in real-time and identify the specific node responsible for the attack, leveraging both temporal and spatial features. Table 9 highlights the features in our input dataset.

### 5.4. Machine Learning and Deep Learning Models Selected for the Development of Our IDS

To ensure the selection of the best-performing model, we evaluated four different machine learning models and one deep learning model, each chosen for its potential effectiveness in identifying anomalies within the CAN bus system. These models were carefully compared based on key performance metrics to determine which would provide the most accurate and reliable detection of DoS attacks. The specific characteristics and advantages of each model are discussed below, highlighting how they contribute to the overall robustness of the IDS.

#### 5.4.1. Logistic Regression

A significant advantage of the logistic regression model [37] lies in its suitability for binary classification problems. In our IDS system, the first step involves detecting the presence of DoS attacks before identifying the specific part of the vehicle under attack. Logistic regression was deemed a promising choice for addressing this initial problem: determining whether a DoS attack is occurring within the vehicle. The objective is to produce a binary outcome, predicting either “Yes” (label 1) or “No” (label 0). Its simplicity and efficiency make it a practical choice for anomaly detection in our IDS system, ensuring seamless integration into vehicle systems.

Logistic regression operates through two key steps: the linear model and the sigmoid function.

The linear model calculates a weighted sum of the input features:

$$z=w_{1}x_{1}+w_{2}x_{2}+\cdots +w_{n}x_{n}+b=w^{⊤}x+b$$

The sigmoid function then maps this result to a probability:

$$\hat{y}=\sigma \left(z\right)=\frac{1}{1+e^{-z}}$$

This allows the model to output probabilities between 0 and 1, which can have included thresholds to ensure binary predictions are produced.

#### 5.4.2. Random Forest Classifier

Another supervised machine learning model chosen for this research is the Random Forest Classifier [38]. Unlike Logistic Regression, which is primarily a classification model, Random Forest is an ensemble learning method that combines the outputs of multiple decision trees. It uses voting for classification and averaging for regression, ensuring optimal performance while reducing the risk of overfitting. These benefits made it an ideal choice for the development of our IDS system.

Random Forest operates in several stages. First, multiple decision trees are built using random subsets of the input data to ensure diversity and robustness. For a given input sample, each tree $T_{k}$ in the forest predicts an output $y_{k}$. In the case of classification tasks, the final output *y* is determined by majority voting:

$$y=\mathrm{mode}(y_{1},y_{2},\ldots ,y_{n}),$$

where $y_{1},y_{2},\ldots ,y_{n}$ are the individual predictions from the *n* trees.

For regression tasks, the final output is computed by averaging the predictions from all trees:

$$y=\frac{1}{n}\sum_{k=1}^{n}y_{k},$$

where $y_{k}$ is the prediction from the *k*-th tree, and *n* is the total number of trees in the forest.

These ensemble methods ensure that Random Forest achieves higher accuracy and robustness compared to individual decision trees. Its ability to handle both classification and regression tasks efficiently made it an ideal selection in the development of the proposed IDS system.

#### 5.4.3. Gradient Boosting

Similar to the Random Forest Classifier, Gradient Boosting [39] is another powerful supervised machine learning model. It is an ensemble learning method capable of performing both classification and regression tasks. Unlike Random Forest, Gradient Boosting builds models sequentially, where each model learns from and corrects the errors of its predecessor. By combining the predictions of weak learners, it creates a strong model capable of making accurate predictions.

For the development of our IDS system, Gradient Boosting offers significant advantages, including high predictive accuracy, customizability, and resistance to overfitting when properly regularized. These characteristics make it a promising choice for enhancing the system’s performance and reliability.

Gradient Boosting combines multiple weak learners (e.g., decision trees) into a strong learner by minimizing a loss function. The process involves iteratively adding a new model $h_{m}\left(x\right)$ to reduce the residual errors of the previous model.

The general prediction for Gradient Boosting is represented as:

$$F_{m}\left(x\right)=F_{m-1}\left(x\right)+\nu h_{m}\left(x\right),$$

where:

- $F_{m}\left(x\right)$: The prediction of the ensemble model at iteration *m*.
- $F_{m-1}\left(x\right)$: The prediction from the previous iteration.
- ν: The learning rate, which controls the contribution of $h_{m}\left(x\right)$.
- $h_{m}\left(x\right)$: The new weak learner added at iteration *m*.

At each iteration, $h_{m}\left(x\right)$ is trained to minimize a loss function L(y,F(x)), which measures the error between the true labels *y* and the predictions F(x). The objective is to solve:

$$h_{m}\left(x\right)=\arg\min_{h}\sum_{i=1}^{n}L\left(y_{i},F_{m-1}\left(x_{i}\right)+h\left(x_{i}\right)\right),$$

where *n* is the number of samples in the dataset.

This sequential optimization process ensures that each new learner focuses on correcting the errors made by its predecessors, resulting in a highly accurate final model.

#### 5.4.4. MultiLayer Perceptron (MLP)

The only deep learning model we selected was the Multilayer Perceptron (MLP) [40], a type of artificial neural network designed for supervised learning tasks. MLP is particularly suited for handling problems with diverse inputs, making it an ideal choice for developing a robust IDS. It excels at predictive analysis by processing input data through multiple layers, where each layer applies transformations based on weights and biases to model complex relationships. Furthermore, MLP adjusts these weights and biases through backpropagation, which minimizes prediction errors and improves accuracy over time.

The computation in an MLP is carried out layer by layer, using weighted sums followed by activation functions. This process, known as the forward pass, is described as follows for each layer *l*:

$$z^{\left(l\right)}=W^{\left(l\right)}a^{\left(l-1\right)}+b^{\left(l\right)},$$

where:

- $z^{\left(l\right)}$ is the input to the activation function at layer *l*,
- $W^{\left(l\right)}$ represents the weights matrix for layer *l*,
- $a^{\left(l-1\right)}$ is the output from the previous layer, and
- $b^{\left(l\right)}$ denotes the bias vector at layer *l*.

Next, the activation function σ is applied to compute the output of layer *l*:

$$a^{\left(l\right)}=\sigma \left(z^{\left(l\right)}\right).$$

The process continues until the final layer generates the output. The accuracy of the MLP improves iteratively through the backpropagation process, where the weights and biases are updated by minimizing the error using a gradient descent algorithm. This adjustment ensures the model learns from the data effectively, enhancing its predictive capabilities.

For all four models selected, the hyperparameters used in their development are summarized in Table 10. These hyperparameters represent the final configurations that were fine-tuned to optimize the architectures of our models, ensuring the best possible results.

### 5.5. Classification Process of the IDS System

The modeling and classification process for detecting DoS attacks and identifying the responsible node involves two main tasks: detecting the occurrence of the DoS attack and classifying which node is responsible for the attack. These two tasks are addressed using different classification strategies, including binary classification for DoS detection and multi-class classification for identifying the node based on its distance from the network controller.

#### 5.5.1. Binary Classification to Detect the Occurrence of DoS Attacks

The first task, DoS attack detection, is framed as a binary classification problem. In this task, the model is trained to predict whether or not a DoS attack is occurring within a given time window. The feature that plays a critical role in determining the presence of a DoS attack is the count of messages with the CAN ID 0. During a DoS attack, the node with ID 0 sends an excessive number of messages, effectively flooding the network due to the CAN bus’s arbitration. By setting a threshold on the number of messages in each time window, the model learns to differentiate between normal and attack behaviors. The binary classification task provides the first layer of defense in identifying potential DoS attacks in real time.

#### 5.5.2. Mutli-Class Classification to Classify the Node Responsible for the Attack

The second task, node identification, is formulated as a multi-class classification problem. Once the DoS attack is detected, the next step is to classify which node is responsible for causing the attack. Each node in the network is assigned a unique distance from the central controller, which serves as an identifier for the node. The goal of the model is to predict the node responsible for the attack based on this distance. In the multi-class classification task, the distance values (or node identifiers) are treated as distinct classes, and the model is trained to predict the class corresponding to the node that initiated the attack. Since multiple nodes can potentially initiate an attack, the model learns to recognize the specific behavior of each node under attack conditions, based on features such as message timing, error counts, and message frequencies. This task is more complex than binary classification, as it requires the model to distinguish between multiple potential sources of the attack, each represented by a different distance value.

#### 5.5.3. Multi-Output Classifier to Predict Both the DoS Attack and the Node Responsible for the Attack

A key aspect of this pipeline is using multi-output classification to jointly predict both the occurrence of a DoS attack and the node responsible for it. Multi-output classification allows the model to predict multiple target variables simultaneously. In this case, the model is tasked with predicting two outputs: the binary label indicating the presence of a DoS attack, and the multi-class label identifying the node responsible for the attack based on its distance. A multi-output classifier is employed to achieve this. This is implemented using the MultiOutputClassifier module from scikit-learn. The MultiOutputClassifier wrapper is utilized to extend traditional machine learning models for multi-output tasks. This classifier builds separate models for each target to allow for the simultaneous training and prediction of multiple outputs. By handling the two tasks of DoS detection and node identification, the multi-output classifier reduces computational overhead and improves the efficiency of the detection process.

The advantage of using a multi-output approach is that the model can leverage shared information between the two objectives. For example, features that are important for detecting a DoS attack (such as the count of the ID 0 messages or timestamp variability) may also provide valuable insights into which node is responsible for the attack. By training the model to predict both outputs simultaneously, the multi-output classifier can take advantage of these shared patterns, leading to better overall performance in both tasks.

Consequently, the modeling and classification process for DoS attack detection and node identification is designed to handle both binary and multi-class classification tasks. By incorporating multi-output classification, the system can simultaneously predict the presence of a DoS attack and identify the responsible node. This approach enhances the model’s efficiency and overall accuracy, making it well-suited for real-time detection scenarios.

## 6. Results

In this section, we describe both the results. The result section presents visualizations and metrics used to evaluate the performance of each model discussed.

### 6.1. Data Visualization Results

Here, we present the results of the data visualization phase, focusing on two key aspects: the raw data before preprocessing and the input data after preprocessing. These visualizations provide valuable insights into the patterns and behaviors within the dataset. They form a critical foundation for understanding the data and guiding the development of our machine learning models.

#### 6.1.1. Data Visualization of the Raw Data Before Preprocessing

**Figure 10:**
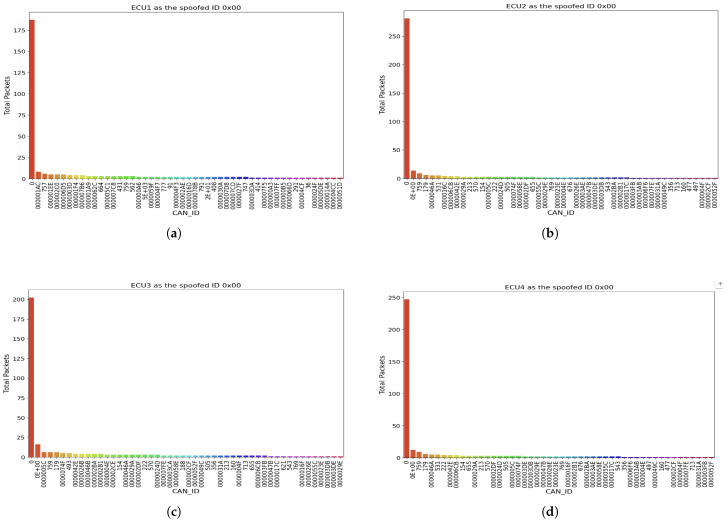
Total count of spoofed packets sent from each node during the 8-h monitoring period. (**a**) Total count of messages when ECU1 was spoofed to ID 0x00. (**b**) Total Count of messages when ECU2 was spoofed to ID 0x00. (**c**) Total Count of messages when ECU3 was spoofed to ID 0x00. (**d**) Total count of messages when ECU4 was spoofed to ID 0x00.

Recall that the data collection phase was divided into two parts: randomized spoofed ID and randomized injection rate. To ensure the collected data accurately represents the presence of a DoS attack, we visualized it using bar charts. Figure 10 illustrates the total count of messages sent during the first phase (randomized spoofed ID).

In this figure, we observe clear evidence of DoS behavior. When spoofed messages were sent from ECU1 (Figure 10a), the total number of packets sent was approximately 187, with the next highest being from ID 0x0E at just 13 packets. This disparity highlights the flood of messages from ECU1. Similarly, Figure 10b demonstrates that when ECU2 acted as the malicious node, the total packets sent reached 281, overwhelming the network. Figure 10c shows that ECU3, as the malicious actor, sent around 202 packets, while Figure 10d highlights ECU4 sending over 247 packets as the spoofed ID.

These visualization results depict the presence of a DoS attack, as the randomized spoofed IDs led to an abnormal spike in packet transmissions, overwhelming the communication channel and disrupting normal operations across the CAN network.

**Figure 11:**
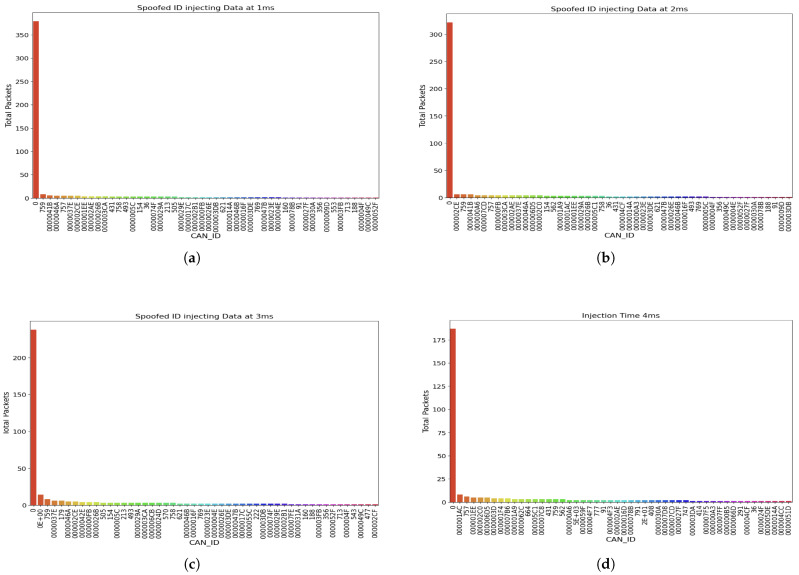
Total count of spoofed packets sent from each node during the 8-h monitoring period at varying injection time. (**a**) Total Count of Spoofed messages when Injection Time is 1 ms. (**b**) Total Count of Spoofed messages when Injection Time is 2 ms. (**c**) Total Count of Spoofed messages when Injection Time is 3 ms. (**d**) Total Count of Spoofed messages when Injection Time is 4 ms.

In the second phase of data collection, we monitored packets from the malicious node spoofed with ID 0x00, ensuring it exploited the arbitration feature of the CAN network. This phase focused on evaluating the impact of flooding at different injection intervals. Figure 11 illustrates the total count of packets sent from the constant spoofed node at varying injection times.

Figure 11a through Figure 11d show the total packets sent when the injection interval was set to 1 ms, 2 ms, 3 ms, and 4 ms, respectively. As expected, we observed that shorter injection intervals resulted in a higher volume of packets. For instance, at a 1 ms injection interval, the total packets sent from the malicious node exceeded 350, whereas at a 4 ms injection interval, the total packets sent decreased to approximately 180.

Additionally, we believe the physical distance of the node to the CAN bus plays a significant role in the number of packets transmitted during a DoS attack. Nodes closer to the bus are likely to generate a higher volume of packets due to reduced latency and more efficient arbitration, further exacerbating the effects of the attack.

**Figure 12:**
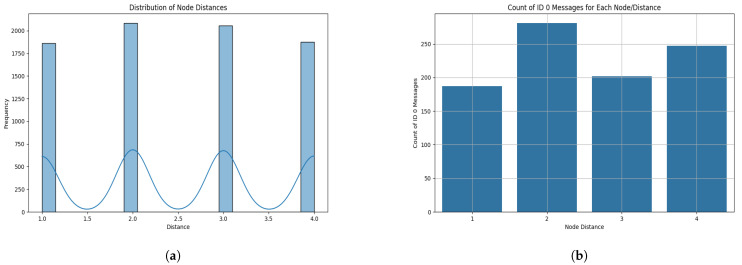
The frequency of distribution from packets sent from all nodes and the total count of all packets from ID 0x00 during the monitoring phase. (**a**) The Frequency distribution of packets across all four nodes. (**b**) The total number of packets sent from ID 0x00 from all four nodes.

We then compile the raw datasets acquired from both phases. The figures in Figure 12 visualize the distribution of node distances and the frequency of ID 0x00 messages for each node, respectively.

Figure 12a illustrates the distribution of nodes by their assigned distances in the dataset. The four distinct distance values (1, 2, 3, and 4) are well represented, with a relatively uniform distribution across all categories. This even distribution ensures that the machine learning models have a balanced dataset to learn from, thereby reducing the risk of bias towards any particular distance class. The slight variations in frequency observed are indicative of inherent data characteristics rather than imbalances in data collection.

Figure 12b demonstrates the count of ID 0x00 messages, which are indicative of potential DoS attacks due to their higher priority in CAN bus arbitration. The bar plot shows that nodes at different distances exhibit varying frequencies of ID 0 messages. Notably, nodes with distances of 2 and 4 show a higher count of ID 0x00 messages compared to nodes with distances of 1 and 3. This pattern suggests that nodes located at these distances may be more prone to generating bursts of high-priority messages, possibly due to their strategic positioning or the simulated attack behavior configured during data collection. These variations in the frequency of ID 0 messages form the basis for the model’s ability to differentiate between normal and anomalous network behavior.

#### 6.1.2. Data Visualization of the Input Data After Preprocessing

**Figure 13:**
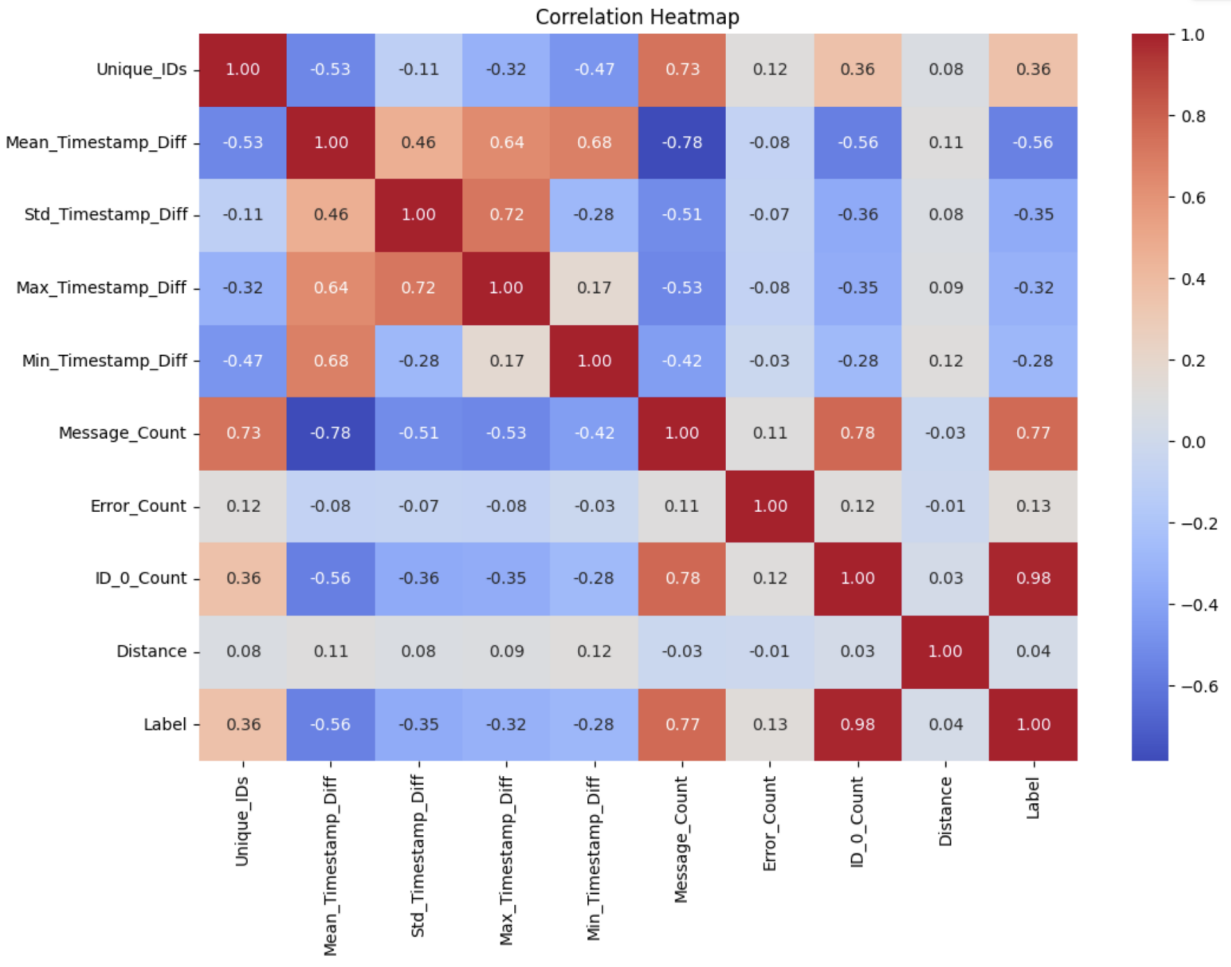
Correlation map showing the features used to train the model.

After acquiring and compiling the raw data, the preprocessing phase included feature engineering steps to introduce noise, simulating more realistic real-world scenarios, and labeling the data which have all being discussed in the previous section. This phase produced the input dataset used to train and evaluate the machine learning models in the development phase of our IDS system. Figure 13 illustrates the correlation heatmap of the input dataset.

In this chart, we observe all the input features and their relationships. Notably, there is a strong correlation between the ID count, node label, and message label. For example, when the ID is 0x00, the label is 1, indicating a DoS attack; otherwise, the label is 0. Negative values in the heatmap indicate no correlation, while positive values signify a high correlation. Strong correlations are observed between features such as message count and unique ID, statistical time analysis, and others. However, no significant correlation is evident between certain features, such as message count and time or label and time.

**Figure 14:**
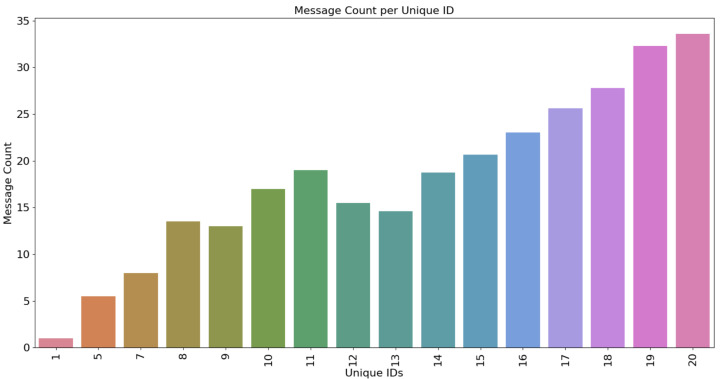
Visual illustration of the connection to the CAN network.

Additionally, we wanted to visualize two key features: the ratio of message count to unique IDs in each monitored 5 ms window. This relationship is critical for understanding how message frequency and diversity relate to potential intrusion activity. Figure 14 provides a visualization of this finding, highlighting the dynamic interplay between these two features.

From the figure, we observe that certain time frames contained little to no messages originating from the malicious node, suggesting periods of normal network activity. However, during other intervals, a clear pattern emerges, indicating a correlation between the number of unique IDs and the message count. Specifically, when a window contains more than 20 unique IDs, the number of messages sent exceeds 35. Conversely, when there are fewer unique IDs we still observe messages being sent, for example when there are 11 unique IDs over 20 messages are sent.

This trend demonstrates the relationship between network activity and unique identifiers, which can signal abnormal behavior indicative of an intrusion. These insights are crucial for understanding the network’s traffic patterns and identifying potential indicators of DoS attacks.

### 6.2. Results

In this subsection, we present the results of the CAN bus intrusion detection experiments. Here, we give a detailed evaluation of the performance of various machine learning models for detecting DoS attacks and identifying the responsible nodes.

#### 6.2.1. Binary Classification Results for Attack Detection

**Figure 15:**
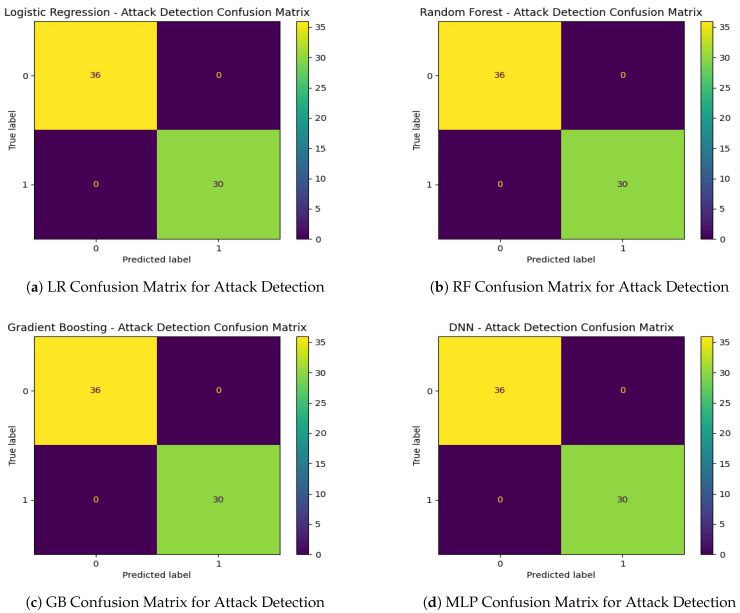
Confusion matrices for the various models in attack detection.

Subsequently, the classification results are evaluated across four different machine learning models: Logistic Regression, Random Forest, Gradient Boosting, and a Multi-Layer Perceptron (MLP). The binary classification task for DoS attack detection showed exceptional performance across all models (Table 11), with perfect accuracy, precision, recall, and F1-scores of 1.00. This consistency highlights the effectiveness of the engineered features in distinguishing attack windows. Confusion matrices (Figure 15a–d) confirm these results, with no false positives or false negatives observed.

To summarize, the consistent values of 1.0 across all models for attack detection in Table 11 indicate that the classification task was straightforward, allowing the models to perfectly distinguish attack scenarios from non-attack scenarios. This could occur under specific conditions or restrictions, such as a clear separation of features where metrics like ID_0_Count or Error_Count have distinct and non-overlapping distributions between attack and non-attack windows, enabling the models to easily learn the decision boundary. Additionally, the threshold for labeling a window as an attack, based on ID_0_Count, has been set to align well with the data distribution, further simplifying classification. The dataset’s balance in terms of attack and non-attack samples could also contribute to this performance, reducing the likelihood of misclassification or overfitting. Furthermore, the binary classification task itself may be inherently less complex if the attack signatures, such as a surge in ID_0_Count, are highly distinguishable. The effectiveness of the feature engineering process, which extracted meaningful characteristics like Message_Count and Error_Count, likely played a key role in capturing the essence of DoS attacks. While these results demonstrate the strength of the models and dataset, further validation under noisier conditions or overlapping feature distributions could ensure the robustness of the approach.

**Figure 16:**
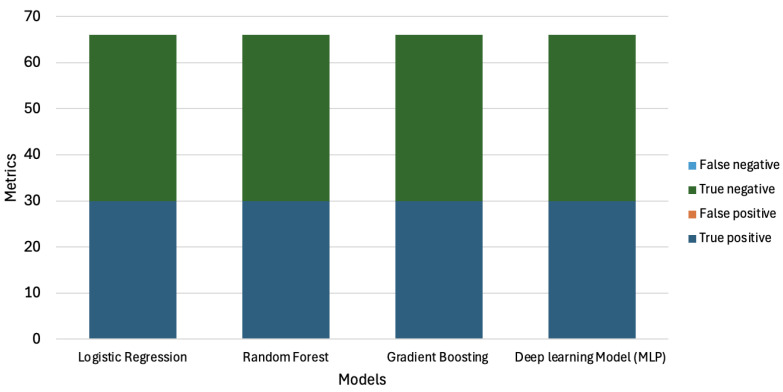
Bar chart showing the confusion matrices for the various models in attack detection. Results showing zero false negative and false positive predictions, indicating that all models accurately identified the presence of an attack when it occurred.

In addition, we have included a visualization of the confusion matrices for each model during the attack detection phase, presented as a stacked bar graph. This illustration is shown in Figure 16. As observed previously, during the first phase of detection, all models demonstrated promising results in detecting DoS attacks, achieving zero false negative and false positive predictions. In this chart, we have illustrated the following components:

- True Positive (TP): The model predicted 1, and the true label was 1 (i.e., a DoS attack is present).
- True Negative (TN): The model predicted 0, and the true label was 0 (i.e., no attack).
- False Positive (FP): The model predicted 1, but the true label was 0 (i.e., an attack was predicted when there was no attack present).
- False Negative (FN): The model predicted 0, but the true label was 1 (i.e., an attack occurred but was not detected by the IDS).

All models used in this research performed well in the first phase, accurately detecting the presence of attacks.

#### 6.2.2. Multi-Class Classification Results for Node Detection

Node detection (multi-class classification) posed greater challenges than attack detection. As shown in Table 12, the Gradient Boosting model achieved the best performance, demonstrating strong pattern recognition for varying node distances. Random Forest and Logistic Regression followed closely, with accuracies of 0.98 and 0.97, respectively. In contrast, the MLP model struggled with node classification, achieving an accuracy of only 0.44.

**Figure 17:**
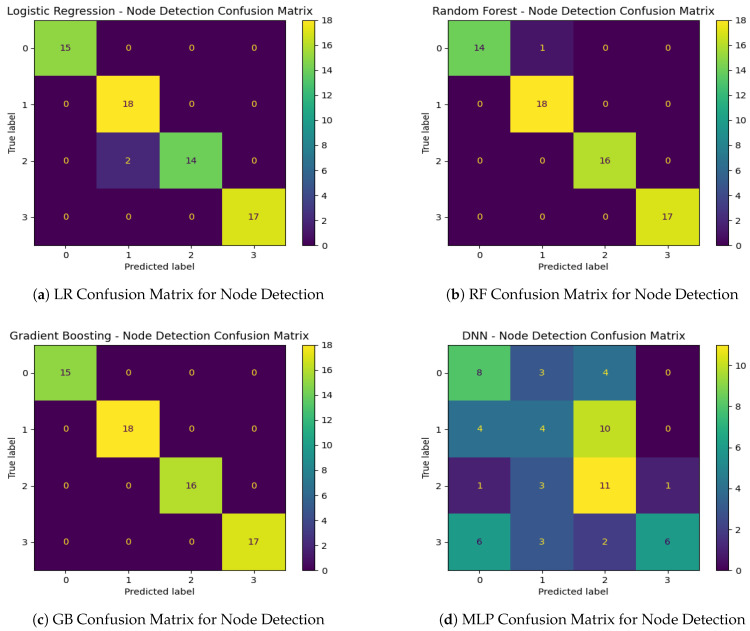
Confusion matrices for the various models in the DoS attack detection.

Figure 17a–d display the confusion matrices for node detection providing further insight into the performance of each model. Logistic Regression, Random Forest, and Gradient Boosting showed clear diagonal dominance, indicating that most samples were correctly classified. However, the MLP model’s confusion matrix reveals significant misclassification across multiple classes, particularly for nodes with distances of 1 and 3, which were often confused with nodes at other distances. This suggests that, while deep learning models like MLP can capture complex patterns, they may require more data or additional tuning to achieve performance levels comparable to tree-based models in this context.

**Figure 18:**
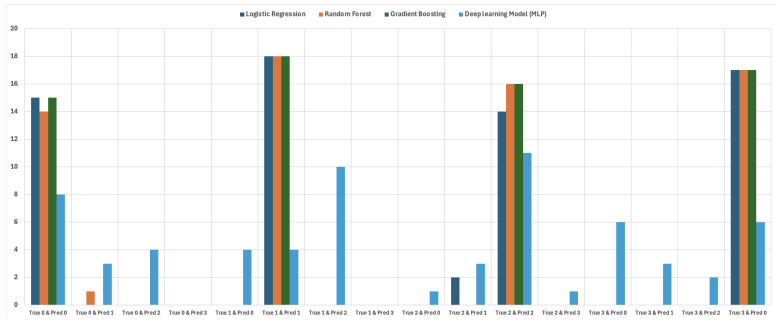
Bar chart showing the confusion matrices for the various models in node detection.

Furthermore, in the node detection phase, we also visualized our confusion matrix findings using a bar chart. Figure 18 illustrates the distribution of each model’s performance in detecting the compromised node within the vehicle. From this figure, we observe that Logistic Regression, Random Forest, and Gradient Boosting accurately identified when Node 2 (label 1) or Node 3 (label 3) were the source of the attack. However, Random Forest and Gradient Boosting misclassified the compromised nodes when Node 1 (label 0) and Node 3 (label 2) were attacked in some cases. Conversely, MLP performed poorly in accurately identifying the node under attack across all cases.

## 7. Discussion

Our results demonstrate the effectiveness of the proposed CANGuard system in detecting anomalies within CAN-enabled vehicles. The system’s performance in binary classification and multi-class node detection tasks provides valuable insights into the strengths and limitations of various machine learning models for intrusion detection in vehicular networks.

### 7.1. Binary Classification Insights

The results for binary classification, where the task was to detect whether a DoS attack was present, were exceptional. All models achieved perfect scores (accuracy, precision, recall, and F1-score of 1.00). These outcomes highlight the robustness of the feature engineering process, which extracted critical temporal and statistical patterns from the raw CAN bus data. The sliding window approach played a pivotal role in capturing fine-grained details about message rates and node activity, ensuring that the models could accurately distinguish between normal and attack states.

This performance indicates that the engineered features are highly effective for attack detection, making the system a promising tool for real-world vehicular IDS deployment. Additionally, the absence of false positives and negatives, as illustrated by the confusion matrices, emphasizes the reliability of the system in detecting attacks without triggering unnecessary alerts or missing actual threats.

### 7.2. Node Detection Challenges

In contrast, the task of node identification (multi-class classification) proved more challenging. While Gradient Boosting emerged as the top-performing model, achieving high accuracy (0.99), the performance varied across other models. Random Forest and Logistic Regression also demonstrated strong capabilities, with accuracies of 0.98 and 0.97, respectively. However, the MLP model struggled significantly, with an accuracy of 0.44, and showed pronounced misclassification errors in the confusion matrix, particularly for nodes at distances 1 and 3.

The disparity in performance suggests that tree-based models, such as Gradient Boosting and Random Forest, are better suited for handling the complexities of node-level detection in this dataset. Their ability to manage feature interactions and capture hierarchical patterns likely contributed to their superior results. On the other hand, the MLP model may require additional tuning, more training data, or architecture adjustments to perform at a comparable level. The results also underscore the importance of dataset balance and feature relevance in multi-class classification tasks.

The binary classification results suggest that CANGuard can serve as a highly reliable first layer of defense in detecting attacks on CAN networks. Its ability to provide accurate attack alerts with no false positives ensures operational safety while minimizing disruptions. For node detection, while Gradient Boosting demonstrates promise, further refinements are needed to ensure consistent and robust performance across all nodes, especially in scenarios with diverse attack patterns and varying data volumes.

The challenges faced by the MLP model point to potential limitations in applying deep learning approaches directly to resource-constrained environments like vehicular systems. This highlights the need for lightweight yet effective models capable of maintaining high performance without excessive computational overhead.

## 8. Conclusions

In this paper, we propose the development and evaluation of CANGuard, an IDS designed to detect DoS attacks in CAN-enabled vehicles. We demonstrate its effectiveness in identifying anomalies within CAN systems by utilizing a hybrid approach that combines anomaly-based and signature-based detection mechanisms. By employing machine learning models and robust preprocessing techniques, CANGuard ensures accurate real-time detection of attacks, including DoS and spoofing, while also identifying compromised nodes. This research underscores the critical role of intelligent IDS solutions in enhancing the security of modern automotive networks, paving the way for safer and more resilient vehicles against emerging cyber threats. Our future work will focus on the development of an advanced security system for CAN bus-enabled vehicles, aimed at effectively preventing potential attacks. This system will leverage robust encryption mechanisms to protect the CAN bus network. By ensuring the integrity and confidentiality of CAN bus communications, we strive to enhance vehicle safety.

## Figures and Tables

**Table 1:** Voltage levels of CAN bus wires in idle (recessive) and active (dominant) states.

CAN Bus Mode/Bit Type	CANH	CANL
Recessive (1)	2.5 V	2.5 V
Dominant (0)	3.75 V	1.25 V

**Table 2:** Components of a frame sent on the CAN bus.

Components	Description	Length of Bits
Start of Frame (SOF)	This is a single dominant bit (0) that shows a message is transmitted on the bus.	1
Identifier (ID)	This is used to show the priority of the message on the bus.	11
Remote Transmission Request (RTR)	This bit shows the type of frame sent, i.e., data frame (0) or recessive frame (1).	1
Control Field	This segment contains two parts; *the IDE (identifier Extension)* bit and *Data Length Code (DLC)*	
- IDE	It shows if the CAN frame is standard or extended. The standard CAN frame contains 11 bits while the extends have an additional 18 bits, making them 29-bit identifiers.	2
- DLC	It indicates the number of data bytes sent on the bus	4
Data	The actual data transmitted by the node on the bus	64
Cyclic Redundancy Check (CRC)	This is used for error detection, providing an avenue for the receiving node to confirm that the data received is correct.	15
Acknowledge (ACK)	The segments consist of two bits, the ACK slot and the ACK Delimiter- a recessive bit that indicates the message was received successfully when it is overwritten. It alerts the node sending the message that the frame was received correctly.	2
End of Frame (EOF)	This signals the end of the frame.	7
Intermission Field	It provides a gap between each frame, ensuring a pause before the next frame is sent to help reduce congestion on the bus.	3

**Table 3:** Critical components for the development and monitoring of the CAN bus system.

Critical Components	Hardware	Purpose
CAN microcontroller	STM Nuclueo-F310RB	Enables reliable message transmission and reception.
CAN controller	Inbuilt into the STM Nuclueo-F310RB	Manages message encoding, transmission, and reception.
CAN transceiver	MCP 2551	Converts signals between the CAN controller and the physical bus.
CAN data logger	CL2000	Logs traffic on the bus
CAN oscilloscope	Picoscope 2000 series	Visualizes and analyzes signal activity on a Controller Area Network.

**Table 4:** Pins Connection for the CAN logger to the CAN network.

CL2000 Connection	CAN Transceiver (MCP2551) Connection
Pin 2	CANL
Pin 3	Ground
Pin 7	CANH
Pin 9	Power supply (5 V)

**Table 5:** Common CAN Bus Attack and their Implications.

Type of Attack	Summary of the Attack	Implication to the Vehicle
DoS	The bus is flooded with messages with high-priority IDs, preventing legitimate communication.	Disrupting legitimate ECU operations, causing possible vehicle malfunction.
Spoofing	False messages are sent on the bus to make it seem like they are from authorized ECUs.	Manipulates the vehicle, thereby altering crucial system operations.
Replay Attack	Attacker captures messages when the car is in operation and then replays it at a later time. that were capt	Leads to unwarranted repeated actions such as turning on or off the door signals.
Fuzzing Attack	This sort of attack is used to discover loopholes in the vehicle by sending random, malformed, or misconstrued messages on the bus.	Vulnerabilities are discovered and the bus could get overloaded with messages causing it to crash.
Message Injection	Messages from unauthorized nodes are sent into the bus.	Prevents messages from legitimate nodes on the bus.
Buss off Attack	This attack targets the ECU by triggering the error response in CAN network which causes it to block a legitimate ECU from sending or receiving messages on the bus until the error is sorted.	Halts operations of an authorized ECU on the bus.
Man-in-the-Middle (MitM)	This attack involves monitoring and capturing sent messages on the bus.	Messages can be modified, blocked thereby altering the normal operations of the vehicle.

**Table 6:** Metrics for Scenario 1 (randomized spoofed IDs).

Metric	Description	Type	Value
Number of ECUs	Total ECUs connected.	Constant	4
Message Type	Type of CAN frame	Constant	Standard Data Frame
Time Interval	Wait time between each message	Constant	5 ms
Spoofed ID	Lowest ID on the bus	Constant	0x00
**Distance from the bus**	**Represented as the ECU name**	**Variable**	**Randomized (ECU1, ECU2, ECU3, ECU4)**
Injection Time	Compromised ECU injection time	Constant	5 ms

**Table 7:** Metrics for Scenario 2 (Randomized Injection Time).

Metric	Description	Type	Value
Number of ECUs	Total ECUs communicating	Constant	4
Message Type	Type of CAN frame	Constant	Standard Data Frame
Time Interval	Wait time between each message	Constant	5 ms
Spoofed ID	Lowest ID on the bus	Constant	0x00
**Injection Time**	**Compromised ECU injection time**	**Variable**	**Randomized (1 ms, 2 ms, 3 ms, 4 ms)**

**Table 8:** Description of the Columns in the raw dataset used for the development of our proposed IDS System.

Column	Description	Relevance	Length (bits)
TimeStamp	Time for each message sent. this is in Unix time format	Selected	32
ID	Message ID to determine priority.	Selected	11
Data Length	The length of the data sent on the bus.	Not Selected	64
Data	The actual data sent.	Not selected	64

**Table 9:** Input dataset.

S/N	Feature
1	Unique ID Count
2	Message Count
3	The Mean timestamp
4	The standard deviation timestamp
5	Difference between the Min and Max timestamps
6	Count of ID 0x00
7	Message Label
8	Node label

**Table 10:** Hyperparameters used to build the models used for the development of the IDS.

Models	Hyperparameters	Values
Logistic Regression	Maximum iteration	1000
	solver	lbfgs
	penalty	l2
	C	1.0
	mutli_class	auto
Random Forest Classifier	n_estimators	100
	criterion	gini
	max_depth	none
	max_features	sqrt
	main_samples_leaf	1
	min_samples_split	3
Gradient Boosting Classifier	learning rate	0.1
	n_estimators	100
	max_depth	3
	min_samples_leaf	1
	min_samples_split	3
	subsample	1.0
	criterion	friedman_mse
MLP	hidden_layer_sizes	(64, 32)
	max_iter	300
	activation	relu
	solver	adams

**Table 11:** Metrics comparison of ML models for attack detection.

Models	Accuracy	Precision	Recall	F1-Score
Logistic Regression	1.00	1.00	1.00	1.00
Random Forest	1.00	1.00	1.00	1.00
Gradient Boosting	1.00	1.00	1.00	1.00
MultiLayer Perceptron	1.00	1.00	1.00	1.00

**Table 12:** Metrics comparison of ML models for node detection.

Models	Accuracy	Precision	Recall	F1-Score
Logistic Regression	0.97	0.97	0.97	0.97
Random Forest	0.98	0.99	0.98	0.98
Gradient Boosting	1.00	1.00	1.00	1.00
MultiLayer Perceptron	0.44	0.50	0.44	0.43

## Data Availability

Data are contained within the article.

## Abbreviations

The following abbreviations are used in this manuscript:

CAN	Controller Area Network
ECU	Electronic Control Unit
ID	Identifier
LR	Logistic Regression
GB	Gradient Boosting
RF	Random Forest
MLP	Multilayer Perceptron
IDS	Intrusion Detection System
DoS	Denial of Service
